# Cesarean scar pregnancy treatment: a case series

**DOI:** 10.1186/s13256-021-03081-0

**Published:** 2021-10-09

**Authors:** Zahra Heidar, Shahrzad Zadeh Modarres, Zhila Abediasl, Arezo Khaghani, Ensieh Salehi, Tayebeh Esfidani

**Affiliations:** 1 Clinical Research Development Center, Mahdiyeh Educational Hospital, Shahid Beheshti University of Medical Sciences, Shishe Gar Khaneh Alley, Fadaian Islam Ave, Shoosh Sq, Tehran, Iran; 2 Fertility and Infertility Research Center, Hormozgan University of Medical Sciences, Bandar Abbas, Iran

**Keywords:** Case series, Cesarean scar pregnancy, Methotrexate

## Abstract

**Background:**

Cesarean scar pregnancy is a complicated and potentially life-threatening type of ectopic pregnancy. This study reports two women with cesarean scar pregnancy who were successfully treated with systemic methotrexate administration, and two other women who needed local re-administration of methotrexate after systemic injection.

**Case presentation:**

Four Iranian pregnant women aged 29–34 years who were between 5  to 7 gestational weeks with cesarean scar pregnancy diagnosis are described. After a single dose of systemic methotrexate injection, the level of serum beta-human chorionic gonadotropin decreased in two of the women, while fetal activity was observed in the other two women. In the latter patients, methotrexate was injected under transvaginal ultrasound guidance into the gestational sac. As a result, the serum beta-human chorionic gonadotropin level first increased and then decreased in these patients. During the follow-up period, all the patients were stable and no complications were observed. Serum beta-human chorionic gonadotropin levels reached the non-pregnancy range from 4 to 9 weeks after treatment.

**Conclusion:**

When diagnosed at early gestation, cesarean scar pregnancy can be treated successfully with methotrexate administration alone. The clinicians should be aware that the beta-human chorionic gonadotropin level may initially increase after methotrexate injection in some patients. However, the final outcome will be promising if the patients remain stable.

## Background

Cesarean scar pregnancy (CSP) is a complicated and potentially life-threatening type of ectopic pregnancy. It is estimated that CSP comprises 0.04–0.05% of all pregnancies [1, 2]. With the increased number of cesarean deliveries and the widespread use of ultrasound (US) in early pregnancy, the incidence of CSP is increasing [2]. Two types of CSP have been defined. Type I (endogenic type) is characterized by a gestational sac implanted in the scar that progresses towards the uterine cavity. Type II (exogenic type) is a deep implantation that mainly grows towards the abdominal cavity. Type II is associated with early uterine rupture and vaginal bleeding [3].

Various medical and surgical treatments including expectant management, systemic methotrexate (MTX), local MTX, dilation and evacuation (D&E), uterine artery embolization, hysteroscopy, and laparoscopy are currently used for the management of CSP [4].

Methotrexate (MTX) is an antimetabolite drug that has been used in the treatment of molar and ectopic pregnancies, including CSP [3]. Here, we describe our experiences of four women with CSP who were successfully treated by systemic MTX administration or a combination of systemic and local MTX administration. In this study, the criteria for the diagnosis of CSP using transvaginal ultrasound (TVU) were an empty uterine cavity, a placenta or pregnancy sac implanted in the cesarean scar site, a pregnancy sac filling the niche of the scar, a thin layer (1–3 mm) of myometrium or its absence between the pregnancy sac and the bladder, a closed cervix and an empty cervical canal, a fetal pole with or without cardiac activity, and the presence of a prominent and at times rich vascular pattern in the area of a cesarean section scar with a positive pregnancy test [3].

## Case presentation

Case 1: A 31-year-old Iranian woman, gravida 2, para 1, with a history of cesarean section (7 years before) was referred to a prenatal clinic for first-trimester screening. She had conceived by an intrauterine insemination (IUI) cycle. Abdominal ultrasonography was performed, and since a gestational sac was located lower than the normal position in her uterine cavity, CSP was suspected. A transvaginal ultrasound revealed a blighted ovum with a 6 mm gestational sac. She was asymptomatic, and her vital signs were stable. Her serum beta-human chorionic gonadotropin (β-hCG) level was 7000 IU/L. After evaluating her renal and liver function tests, one dose of systematic MTX (50 mg/m^2^) was administered. The serum β-hCG level decreased from 7000 to 4900 IU/L after 4 days. During the follow-up period, the β-hCG level decreased continuously. After 1 week, vaginal bleeding occurred and the remnants of pregnancy were expelled. Four weeks after MTX administration, the serum β-hCG level reached the non-pregnancy range. No complications occurred during the treatment.

Case 2: A 34-year-old Iranian woman with a history of cesarean section (8 years before) was referred to our hospital for prenatal care. She had conceived with an *in vitro* fertilization (IVF) cycle. With abdominal ultrasound imaging, CSP was suspected due to the improper location of the gestational sac. This was confirmed using TVU. The gestational sac was measured to be 10 mm, showing that the gestational age was 6 weeks. The serum β-hCG level was 19,000 IU/L. Her vital signs were stable, and she was asymptomatic. The patient was hospitalized for management, and liver and renal function tests were performed. The patient was counseled for medical and surgical management and opted for medical management. After obtaining informed consent, one dose of 60 mg systemic MTX was administered and repeated 48 hours later. Four days later, her serum β-hCG level reached 29,000 IU/L, and cardiac activity was observed on TVU. For this reason, 0.3 cc KCl was injected into the embryo. Then, 30 mg of MTX was injected transvaginally into the gestational sac. Twenty-four hours later, the serum β-hCG level increased to 45,000 IU/L. As she remained clinically stable, we decided to follow her up with serial serum β-hCG measurements. Four days later, her serum β-hCG level reached 32,000 IU/L and she was discharged. Her serum β-hCG values were measured weekly. Nine weeks later, the serum β-hCG reached the non-pregnancy range. Side effects of MTX administration were not observed. Serial ultrasound assessments revealed a persistent 15 mm mass (including blood clots and fragments of decidualized tissue and secretory endometrium) in the cesarean scar, which was removed by hysteroscopy.

Case 3: A 29-year-old Iranian woman, gravida 3, with a history of one miscarriage in the sixth week of pregnancy and a history of cesarean section (4 years before) was referred to our hospital for routine pregnancy ultrasonography. The gestational age based on the last menstrual period was 7 weeks. An abdominal ultrasound scan suggested CSP with a fetal pole lower than the normal position with a gestational age of 6 weeks. TVU revealed CSP with a blighted ovum, and the β-hCG level was 11,000 IU/L. The patient did not have vaginal bleeding, abdominal pain, or discomfort. After consultation with the patient and her husband about the management, her vital signs were checked and kidney and liver function tests were performed. Afterward, she received 50 mg/m^2^ of systemic MTX. Four days later, the β-hCG level reached 10,000 IU/L. Seven days later, the β-hCG level decreased to 7500 IU/L. The patient had spotting but remained stable. The β-hCG level was checked weekly. In the sixth week, the β-hCG level decreased to less than 10 and the pregnancy remnants (approximately 2 cm) were removed by hysteroscopy.

Case 4: A 33-year-old Iranian woman, gravida 3, para 2, live 2, with a history of cesarean section (6 years before) whose last menstrual period was 5 weeks before was admitted to our hospital owing to the diagnosis of CSP using TVU. The gestational age was 5 weeks and 5 days. Her vital signs were stable, and she had no abdominal pain and no vaginal bleeding symptoms.

The β-hCG level was 3546 IU/L. After consulting with the patient, she was hospitalized. After performing kidney and liver function tests, she received 50 mg/m^2^ systemic MTX. Four and seven days after MTX injection, the β-hCG level reached 4800 IU/L and 5750 IU/L, respectively. The second dose (70 mg) was repeated 7 days after the first dose. Four days later (after the second dose), the β-hCG level became 6500 IU/L. Since fetal heart activity was observed on vaginal ultrasonography, MTX (70 mg) was administered transvaginally. Two days after transvaginal MTX injection, the β-hCG level increased to 7100 IU/L. Four days after transvaginal MTX administration, the β-hCG level decreased to 4100 IU/L. Afterward, the patient was monitored for her stability and absence of symptoms. Two days later, mild vaginal bleeding occurred. The β-hCG level was checked weekly. The patient was discharged in good general condition and was advised to check her β-hCG level weekly. Five weeks later, the β-hCG level reached the non-pregnancy range. TVU showed no sac, and the remnants of pregnancy (including fragments of decidualized tissue and secretory endometrium) were removed by hysteroscopy (Table 1).

**Table 1:** Patients’ demographic, clinical characteristics, and outcomes

Characteristics	Case 1	Case 2	Case 3	Case 4
Age (years)	32	34	29	33
Previous cesarean section	Yes	Yes	Yes	Yes
History of curettage	No	No	Yes	No
Gestational age	6 weeks	6 weeks	6 weeks	5 weeks and 5 days
Size of the sac (mm)	10	14	14	11
Initial β-hCG (IU/L)	7000	19,000	11,000	3546
Primary treatment	Systemic MTX	Systemic MTX	Systemic MTX	Systemic MTX
β-hCG level 4 days after treatment (IU/L)	4900	29,000	10,000	4800
Second treatment	No	Yes	No	Yes
β-hCG level 4 days after second treatment (IU/L)	–	32,000	–	6500
Time to reach negative β-hCG	4 weeks	9 weeks	6 weeks	5 weeks

*MTX* Methotrexate, *β-hCG* Beta-human chorionic gonadotropin

## Discussion and conclusions

Cesarean scar pregnancy (CSP) is defined by the development of a gestational sac in the myometrium of a previous cesarean scar [5]. Although the exact pathology underlying CSP is still unclear, impaired healing of the cesarean section wound can predispose women to CSP. Further, CSP may result from a defect in the endometrium caused by trauma [6]. The symptoms of CSP are unspecific, and one-third of cases are asymptomatic [7]. In this report, all patients were asymptomatic and were diagnosed on first-trimester ultrasound. Therefore, early routine ultrasound at the beginning of pregnancy is recommended in pregnant women with a history of previous cesarean section. The most common diagnosis techniques of CSP are abdominal ultrasonography, TVU, and color Doppler. The sensitivity of TVU is reported to be 84% [8]. In all women of this study, the size and location of the gestational sac was determined using TVU.

The treatment options for CSP are medical, surgical, or a combination of them. Since women with CSP are of reproductive age and would like to preserve their fertility, the selected treatment should retain their fertility. Local or systemic administration of MTX is one of the most popular treatments for CSP because of its quick response and fewer side effects. The present study demonstrated that a single dose of systemic MTX administration resulted in a safe treatment without further intervention in two patients with CSP. However, in two other patients, the β-hCG level increased following a single dose of systemic MTX administration. Since the serum β-hCG level was not decreased as expected, multiple doses of MTX as a combination of systemic and local (injected into the sac) MTX were used. Levin *et al*. reported that 29/36 cases (80.6%) were treated successfully by systemic injection of MTX, while the other 19.4% were treated with a combination of systemic and local (that is, intra-sac) MTX administration [9]. Bodur *et al*. concluded that a primary systemic MTX administration was effective for a cesarean scar ectopic pregnancy before 8 weeks of gestational age, a β-hCG concentration of ≤ 12,000 mIU/ml, and negative embryonic cardiac activity [10]. In the present study, all of the mentioned criteria were present in the two patients who were successfully treated with a primary systemic MTX administration. In this regard, another study demonstrated that the failure of the MTX treatment was associated with a high β-hCG level, advanced pregnancy, and deep implantation. Moreover, it was found that the resolution of pregnancy was relatively faster in the two patients that received a single dose of systemic MTX compared with the other two patients. A clinical trial study indicated that a single dose of systemic MTX had an equally successful rate compared with local MTX administration (67.3% versus 69.2%, respectively). However, the decline of the serum β-hCG level and pregnancy disappearance were faster in the systemic group.

It has been reported that 25% of patients need additional treatment owing to increased β-hCG levels and heart activity following MTX administration. The β-hCG level was an important prognostic factor in treatment failure [11]. However, no specific β-hCG level has been determined to guarantee treatment success [12]. In our observations, two patients showed higher β-hCG levels following the first dose of systemic MTX administration. This indicated primary treatment failure. Therefore, an additional injection into the sac (transvaginal MTX injection) was needed.

It is important to know that the serum β-hCG level, the gestational sac volume, and vascularization may temporarily increase after a combined local and systemic MTX administration for the treatment of CSP [13]. In accordance with the findings of the current study, Timor-Tritsch *et al*. also reported that the β-hCG level increased after combined local and systemic MTX administration [13]. Another study found that the mean serum β-hCG level tended to increase in 38 women with ectopic pregnancy in the first 4 days after systemic MTX injection [14]. In a review of eight patients with CSP, Yamaguchi *et al*. reported that the β-hCG level first increased and then decreased [15]. Therefore, the increase of the β-hCG level at the beginning of local MTX injection is probably not worrying if the patient remains stable.

In conclusion, when diagnosed at early gestation, cesarean scar pregnancy can be successfully treated with medical management alone. Nevertheless, in advanced gestation, it usually leads to excessive vaginal bleeding, higher β-hCG levels, and risk of failure of medical management alone. The clinicians should be aware that the β-hCG level, as a prognostic factor for treatment success, may initially increase after MTX injection in some patients. However, the final outcome will be promising if the patients remain stable.

## Data Availability

The datasets supporting this article are included within the article

## Abbreviations

CSP: Cesarean scar pregnancy
MTX: Methotrexate
β-hCG: Beta-human chorionic gonadotropin
IUI: Intrauterine insemination
TVU: Transvaginal ultrasound
IVF: *In vitro* fertilization

## References

[1] Maymon R, Halperin R, Mendlovic S, Schneider D, Herman A. Ectopic pregnancies in a Caesarean scar: review of the medical approach to an iatrogenic complication. Hum Reprod Update. 2004;10(6):515–23. 10.1093/humupd/dmh042 15375087

[2] Baradwan S, Khan F, Al-Jaroudi D. Successful management of a spontaneous viable monochorionic diamniotic twin pregnancy on cesarean scar with systemic methotrexate: a case report. Medicine. 2018. 10.1097/MD.0000000000012343. PMC6155970 30212987

[3] Le Minh Tam TK, Linh TM, Thu PTM, Khanh TV, Anh NTK, Nguyen NTT, *et al*. Outcome of cesarean scar pregnancy treated with local methotrexate injection. Nagoya J Med Sci. 2020;82(1):15. 10.18999/nagjms.82.1.15 PMC7103867 32273628

[4] Junaid D, Chaudhry S, Usman M, Hussain R. Caesarean scar ectopic pregnancy: a case series. Pak J Med Dent. 2018;7(3):4.

[5] Wang S, Beejadhursing R, Ma X, Li Y. Management of Caesarean scar pregnancy with or without methotrexate before curettage: human chorionic gonadotropin trends and patient outcomes. BMC Pregnancy Childbirth. 2018;18(1):1–6. 10.1186/s12884-018-1923-x PMC6032533 29973177

[6] Xiao J, Zhang S, Wang F, Wang Y, Shi Z, Zhou X, *et al*. Cesarean scar pregnancy: noninvasive and effective treatment with high-intensity focused ultrasound. Am J Obstetr Gynecol. 2014;211(4):356 e1-356 e7. 10.1016/j.ajog.2014.04.024 24769010

[7] Gonzalez N, Tulandi T. Cesarean scar pregnancy: a systematic review. J Minim Invasive Gynecol. 2017;24(5):731–8. 10.1016/j.jmig.2017.02.020 28268103

[8] Pristavu A, Vinturache A, Mihalceanu E, Pintilie R, Onofriescu M, Socolov D. Combination of medical and surgical management in successful treatment of caesarean scar pregnancy: a case report series. BMC Pregnancy Childbirth. 2020;20(1):1–10. 10.1186/s12884-020-03237-8 PMC7557042 33050911

[9] Levin G, Zigron R, Dior UP, Gilad R, Shushan A, Benshushan A, *et al*. Conservative management of Caesarean scar pregnancies with systemic multidose methotrexate: predictors of treatment failure and reproductive outcomes. Reprod Biomed Online. 2019;39(5):827–34. 10.1016/j.rbmo.2019.05.015 31530445

[10] Bodur S, Özdamar Ö, Kılıç S, Gün I. The efficacy of the systemic methotrexate treatment in caesarean scar ectopic pregnancy: a quantitative review of English literature. J Obstet Gynaecol. 2015;35(3):290–6. 10.3109/01443615.2014.954101 25259651

[11] Lipscomb GH, Givens VM, Meyer NL, Bran D. Comparison of multidose and single-dose methotrexate protocols for the treatment of ectopic pregnancy. Am J Obstet Gynecol. 2005;192(6):1844–7. 10.1016/j.ajog.2004.12.061 15970826

[12] Menon S, Colins J, Barnhart KT. Establishing a human chorionic gonadotropin cutoff to guide methotrexate treatment of ectopic pregnancy: a systematic review. Fertil Steril. 2007;87(3):481–4. 10.1016/j.fertnstert.2006.10.007 17173905

[13] Timor-Tritsch IE, Monteagudo A, Santos R, Tsymbal T, Pineda G, Arslan AA. The diagnosis, treatment, and follow-up of cesarean scar pregnancy. Am J Obstetr Gynecol. 2012;207(1):44 e1-44 e13. 10.1016/j.ajog.2012.04.018 22607667

[14] Saraj AJ, Wilcox JG, Najmabadi S, Stein SM, Johnson MB, Paulson RJ. Resolution of hormonal markers of ectopic gestation: a randomized trial comparing single-dose intramuscular methotrexate with salpingostomy. Obstet Gynecol. 1998;92(6):989–94. 10.1016/s0029-7844(98)00324-x 9840564

[15] Yamaguchi M, Honda R, Uchino K, Tashiro H, Ohba T, Katabuchi H. Transvaginal methotrexate injection for the treatment of cesarean scar pregnancy: efficacy and subsequent fecundity. J Minim Invasive Gynecol. 2014;21(5):877–83. 10.1016/j.jmig.2014.03.024 24743111

